# Grafting Enhances Pepper Water Stress Tolerance by Improving Photosynthesis and Antioxidant Defense Systems

**DOI:** 10.3390/antiox10040576

**Published:** 2021-04-08

**Authors:** Yaiza Gara Padilla, Ramón Gisbert-Mullor, Lidia López-Serrano, Salvador López-Galarza, Ángeles Calatayud

**Affiliations:** 1 Centro de Citricultura y Producción Vegetal, Instituto Valenciano de Investigaciones Agrarias, Departamento de Horticultura, CV-315, Km 10,7, Moncada, 46113 Valencia, Spain; padilla_yai@gva.es (Y.G.P.); lopez_lid@gva.es (L.L.-S.); 2 Departamento de Producción Vegetal, CVER, Universitat Politècnica de València, Camí de Vera s/n, 46022 Valencia, Spain; ragismul@etsiamn.upv.es (R.G.-M.); slopez@upv.es (S.L.-G.)

**Keywords:** *Capsicum annuum*, grafted plants, gas exchange, drought, oxidative stress, rootstock

## Abstract

Currently, limited water supply is a major problem in many parts of the world. Grafting peppers onto adequate rootstocks is a sustainable technique used to cope with water scarcity in plants. For 1 month, this work compared grafted peppers by employing two rootstocks (H92 and H90), with different sensitivities to water stress, and ungrafted plants in biomass, photosynthesis, and antioxidant response terms to identify physiological–antioxidant pathways of water stress tolerance. Water stress significantly stunted growth in all the plant types, although tolerant grafted plants (variety grafted onto H92, Var/H92) had higher leaf area and fresh weight values. Var/H92 showed photosynthesis and stomata conductance maintenance, compared to sensitive grafted plants (Var/H90) and ungrafted plants under water stress, linked with greater instantaneous water use efficiency. The antioxidant system was effective in removing reactive oxygen species (ROS) that could damage photosynthesis; a significant positive and negative linear correlation was observed between the rate of CO_2_ uptake and ascorbic acid (AsA)/total AsA (AsA_t_) and proline, respectively. Moreover, in Var/H92 under water stress, both higher proline and ascorbate concentration were observed. Consequently, less membrane lipid peroxidation was quantified in Var/H92.

## 1. Introduction

Abiotic stresses pose a huge threat for crops by limiting their growth and development, which eventually leads to poor productivity and low yields [1,2,3]. One abiotic stress is water limitation, which is considered a major problem because of its high intensity and time span, and because it causes significant crop losses worldwide every year [4,5,6,7]. Besides, many crops are now cultivated in areas where climate conditions are not always ideal, and precipitation may periodically go below optimal levels and lead to water scarcity [8].

Many kinds of approaches have been tested to deal with this threat, including agricultural practices focused on irrigation management, but also the introgression of drought resistance traits by molecular breeding. On the one hand, agricultural practices are not always suitable, given the peculiarities of each growing area and the unpredictability of drought periods. On the other hand, molecular and conventional breeding strategies have faced many difficulties when assessing drought tolerance because it is achieved by combining quantitative characters. These, in turn, are controlled by many minor genes with additive effects [2,4,5,6,9]. Alternatively, Genetically Modified plants have been developed, but these organisms are closely regulated and, like molecular breeding findings, they need to be validated in the field to prove their usefulness [10].

Given all these different approaches, vegetable grafting emerges as a sustainable effective technique, particularly in Solanaceae and Cucurbitaceae species, to overcome water deficit and other abiotic stresses by preventing harmful impacts on shoots [5,11,12,13,14]; because rootstock (root) performance stands out over scion (shoot) behavior when stress affects soil [15].

Several studies have confirmed positive physiological traits when employing tolerant rootstocks under water starvation conditions compared to ungrafted, self-grafted or plants grafted on susceptible rootstocks [16,17,18,19,20]. Photosynthesis is one of the first metabolic processes to be affected by drought [21,22]. Grafting has been identified as an effective tool for increasing CO_2_ assimilation and intrinsic water use efficiency (A_N_/E, WUE) [12,13] when tolerant rootstocks are employed. The increased WUE in tolerant grafted plants, compared to ungrafted plants or using sensitive rootstocks, is often related to a higher net CO_2_ assimilation rate and less transpiration, and is joined with stomata regulation [23,24,25]. However, a drop in WUE has also been observed in two tomato landraces grafted onto commercial tomato rootstocks, which has been associated with a more marked increase in stomatal conductance, linked with minor increases in net photosynthesis [26,27]. These differential WUE patterns indicate that WUE performance can depend not only on the rootstock, but also on the scion used, and mainly on water stress severity [28,29]. Generally with mild and moderate water stress, the decline in A_N_ can be less severe than the drop in g_s_, which causes WUE to increase. These results imply that stomatal limitations are responsible for reducing A_N_ under drought stress, which is associated with a down-regulated photosynthetic metabolism [22,25,30]. Nevertheless, with severe water stress A_N_ values drop more than g_s_ values and, consequently, WUE usually lowers. This situation implies the existence of stomatal, but mainly non-stomatal limitations (inhibition of metabolic processes) connected with photosynthesis damage, which thus affects physiological adaptation to water stress [18,22].

Maintaining photosynthesis under water limitation levels is essential because its suppression could increase excitation energy excess and electron flux to O_2_; which would result in photo-oxidative stress by reactive oxygen species (ROS) overproduction [31,32]. ROS damage proteins and nucleic acids by causing membrane dysfunction, followed by lipid peroxidation and enzymes inactivation [33]. ROS detoxification is most important in any defense mechanism, this is why plants possess a complex antioxidant system that includes non-enzymatic molecules (e.g., ascorbate, proline, phenols, tocopherols [34]), but also antioxidant enzymatic components (e.g., catalase, superoxide dismutase, peroxidase, and components of the ascorbate–glutathione cycle [35]).

Increasing antioxidant system activities is necessary to enhance plant protection and for reaching tolerance below non optimal water levels [8]. In recent years, more attention has been paid to understand the antioxidative system in plants under water deficit [36], but less research has been performed in grafted plants to identify potential antioxidative mechanisms of drought tolerance. These studies have been performed mainly in tomato, watermelon and cucumber grafted plants [5], with very few reports available on antioxidants regulation in pepper-grafted plants [16,20].

Pepper is one of the most important crops grown in the Mediterranean climate, where water scarcity is a relevant constraint [16]. Nevertheless, commercial rootstocks are not usually employed in pepper plants as they do not provide enough benefits [37,38]. 

Our previous studies performed classical breeding assays with pepper accessions (*C. annuum* × *C. annuum*) that resulted in hybrids with more uniform germination, vigor, and growth. These hybrids were tested and screened under water stress conditions based on photosynthetic parameters, leading to identify both tolerant and sensitive hybrids under water stress (unpublished data). Of these, one tolerant rootstock (H92) with higher CO_2_ assimilation and one sensitive rootstock (H90) were chosen to test the hypothesis herein posed.

The aim of the present study is to determine whether the maintenance of photosynthesis observed in the tolerant hybrid rootstock (H92) under water stress is associated with the protection of the photosynthetic apparatus, mediated by the activation of the antioxidant system components. For this purpose, several parameters, such as biomass, photosynthetic parameters, ascorbate, proline, catalase activity, and lipid peroxidation were determined in grafted plants using tolerant and sensitive rootstocks and ungrafted plants, grown both in water stress and control conditions.

## 2. Materials and Methods

### 2.1. Plant Material

Two *Capsicum annuum* hybrids (codes H92 and H90, tolerant and sensitive to water stress, respectively), obtained through traditional breeding by crossing pepper accessions in previous studies [39,40,41], were selected from a pool of 11 hybrids to be used as rootstocks in the present study. Pepper landrace “Sueca” (Var) was employed as scion and as ungrafted plants. Seeds were sown in March 2020 in 104-hole trays with enriched substrate under greenhouse conditions. Two months after germination, Var was grafted by the tube-grafting method onto the studied hybrid rootstocks. Three weeks after grafting, plants were transplanted in 6-L pots with coconut coir fiber (Cocopeat, Projar Co., Valencia, Spain) in a polyethylene greenhouse at the Valencian Institute of Agriculture Research (IVIA; Valencia, Spain). During the experimental period, temperature ranged from 20 °C to 30 °C, with 40–80% relative humidity (RH). Twelve plants per plant combination were used. 

After allowing pepper plants to adapt to the greenhouse for 1 week, they were distributed for two treatments (six plants per treatment). Under the water stress conditions, water irrigation was lowered to 55% of total irrigation under the control conditions, which was calculated based on the weekly crop evapotranspiration (ETc). The number of irrigations was scheduled to maintain drainage between 20% and 30%. Plants were irrigated with Hoagland’s no. 2 nutrient solution containing (all in mM): 14 NO_3_^−^, 1.0 H_2_PO_4_^−^, 2.0 SO_4_^2−^, 1.0 NH_4_^+^, 6.0 K^+^, 4.0 Ca^2+^, and 2.0 Mg^2+^. Micronutrients were also provided (all in μM): 15 Fe^2+^, 10 Mn^2+^, 5 Zn^2+^, 30 B^3+^, 0.75 Cu^2+^, and 0.6 Mo^6+^) [42]. Electrical conductivity was 1.9 dSm^−1^ and pH was 6.7. To apply the same fertilizer doses in the control and water stress treatments, nutrient solution was applied to both treatments when irrigation to stressed plants was administered. To meet irrigation water requirements, only water was applied to the other irrigation events in the non-stressed treatment.

All of the measurements were taken 10 days after treatment (10 DAT) started, 20 DAT, and 30 DAT. During each measurement event, four measurements (one plant/replication) per plant type and treatment combination were taken. Photosynthetic parameters and physiological analyses were measured on the third or fourth leaves from the apex. For the physiological analysis, leaves were previously frozen with liquid nitrogen and stored at −80 °C. Samples were ground by a mixer mill (MM400, Retsch, Hann, Germany) with liquid nitrogen to prevent melting.

### 2.2. Water Relations

Osmotic potential of leaf sap (Ψs, in MPa) and relative water content (RWC) were measured at 30 DAT in leaves. RWC was determined by weighing leaves before and after a 24 h rehydration process performed with distilled water, obtaining the fresh weigh (FW) and turgid weight (TW), respectively. To obtain the dry weight (DW), leaves were dried at 65 °C for 72 h and then weighed. RWC was determined as: RWC (%) = (FW − DW)/(TW − DW) × 100.

The Ψs was measured by an osmometer (Digital Osmometer, Wescor, Logan, UT, USA). Leaves were detached, placed inside 1 mL tubes, and quickly frozen at −20 °C. After melting, sap was collected by centrifugation for 1 min at 9.000 rpm in 1.5 mL tubes. Osmolyte content of leaf sap (mmol kg^−1^) was converted into MPa by the Van’t Hoff equation [18].

### 2.3. Gas Exchange Measurements 

The CO_2_ assimilation rate (A_N_, µmol CO_2_ m^−2^ s^−1^), stomatal conductance to water vapor (g_s_, mol H_2_O m^−2^ s^−1^), substomatal CO_2_ concentration (C_i_, µmol CO_2_ mol^−1^ air) and transpiration rate (E, mmol H_2_O m^−2^ s^−1^) were measured by a portable LI-COR 6400 infrared gas analyzer (Li-Cor Inc., Lincoln, NE, USA). The A_N_/E parameter was calculated as instantaneous WUE. Measurements were taken under saturating light conditions (1000 µmol quanta m^−2^ s^−1^), 400 µmol CO_2_ mol^−1^ of reference CO_2_, at 27 °C (27 ± 2 °C) and 75% RH. For each measurement time, data were collected on two consecutive days from 09:00 h to 11:00 h (UT + 01:00 h).

### 2.4. Ascorbate Metabolism 

Ascorbic acid (AsA), dehydroascorbate (DHA) and total AsA (AsA_t_ = AsA + DHA) were determined in parallel according to [43] with some variations. First 0.4 g of sample was mixed with 80% (*w*/*v*) trichloroacetic acid (TCA) and centrifuged for 5 min at 15,000× *g* and 4 °C. Then 50 µL of the supernatant were mixed with 150 µL of 0.2 M phosphate buffer (pH 7.4) and 50 µL of distilled H_2_O_2_ for the AsA determination. For AsA_t_, 50 µL of the supernatant were mixed with 50 µL of 10 mM dithiothreitol (DTT) and 100 µL of 0.2 M phosphate buffer (pH 7.4), and incubated in a water bath at 42 °C for 15 min. Then 50 µL of 0.5% (*w*/*v*) N-ethylmaleimide (NEM) were added and samples were incubated for 1 min at room temperature. Both AsA and AsA_t_ tubes were mixed with 250 µL of 10% (*w*/*v*) TCA, 200 µL of 42% (*v*/*v*) H_3_PO_4_, 200 µL of 4% (*w*/*v*) 2,2′-dipyridyl and 100 µL of 3% (*w*/*v*) FeCl_3_, and incubated in a water bath for 40 min at 42 °C. Absorbance was recorded at 525 nm in both cases. The DHA concentration was determined as: AsA_t_ − AsA.

### 2.5. Catalase Activity

Catalase enzyme activity (EC 1.11.1.6) was measured as in [44], but with modifications: 0.5 g of the sample was mixed with 2.5 mL of 10 mM potassium phosphate buffer (with 1.27 mM EDTA and pH 7) and 2.5% (*w*/*v*) polyvinylpolypyrrolidone (PVPP), and was centrifuged for 30 min at 10,000× *g* and 4 °C. The supernatant was conserved under ice conditions until measurements were taken. To start the reaction, 1960 µL of 50 mM potassium phosphate (pH 7) were mixed with 20 µL of sample and 20 µL of H_2_O_2_. Reduction of H_2_O_2_ by catalase activity was monitored spectrophotometrically at 240 nm for 4 min at room temperature. U (µmol/min) g^−1^ FW was calculated using the Lambert–Beer equation with the H_2_O_2_ extinction coefficient (ε = 39.4 mM^−1^ cm^−1^).

### 2.6. Lipid Peroxidation Analysis

Lipid peroxidation was determined by the malondialdehyde (MDA) procedure using the thiobarbituric acid (TBA) reaction according to [45], and modified by [46]: 0.1 g of sample was mixed with 2 mL of 0.1% (*w*/*v*) TCA and centrifuged for 5 min at 10,000× *g* and 4 °C. Later, 1 mL of supernatant was mixed with 2 mL of reaction buffer (20% TCA + 0.5% TBA) and samples were incubated for 30 min at 95 °C in a water bath. The non-specific background absorbance reading at 600 nm was subtracted from the specific absorbance reading at 532 nm.

### 2.7. Proline Determination

Proline quantification was performed as reported in [47] with slight differences: 0.2 g of the sample was mixed with 1.5 mL of 3% sulfosalicylic acid and centrifuged for 5 min at 14,000 rpm and room temperature. Next, 0.6 mL of glacial acetic acid and 0.7 mL of ninhydrin reagent (40 mL of 6 M phosphoric acid mixed with 2.5 g ninhydrin previously blended in 600 mL of glacial acetic acid) were added to 70 µL of the supernatant, and samples were heated for 1 h at 100 °C. Absorbance measures were taken at 520 nm and interpolated on a standard curve performed with proline. 

### 2.8. Plant Biomass and Leaf Area Determination

The biomass parameters were measured at the end of the experiment (30 DAT). Leaves and stems were weighed and the number of leaves per plant was recorded. The total leaf area was measured with a LI-3100 Area Meter (Li-COR Inc., Lincoln, NE, USA). Later leaves and stems were exposed to dry heat (for 72 h at 70 °C) in a laboratory oven and dry weight (DW) was recorded.

### 2.9. Statistical Analysis

The results of all the parameters were subjected to a two-way analysis of variance (ANOVA) with Statgraphics Centurion 18 (Statgraphics Technologies Inc., The Plains, VA, USA) after including two factors, namely plant type (PT) and treatment (T), and by considering their interaction. The physiological parameters were analyzed separately for each measurement time (10, 20, and 30 DAT). The means of all the parameters were compared by Fisher’s least significance difference (LSD test) at *p* < 0.05. No significant differences were found in the replicates for each measured parameter. The correlation analyses between A_N_ and AsA/AsA_t_ and A_N_ and proline were performed with the above-mentioned Statgraphics software and the correlation coefficient (r) was obtained.

## 3. Results

### 3.1. Water Relations

**Figure 1:**
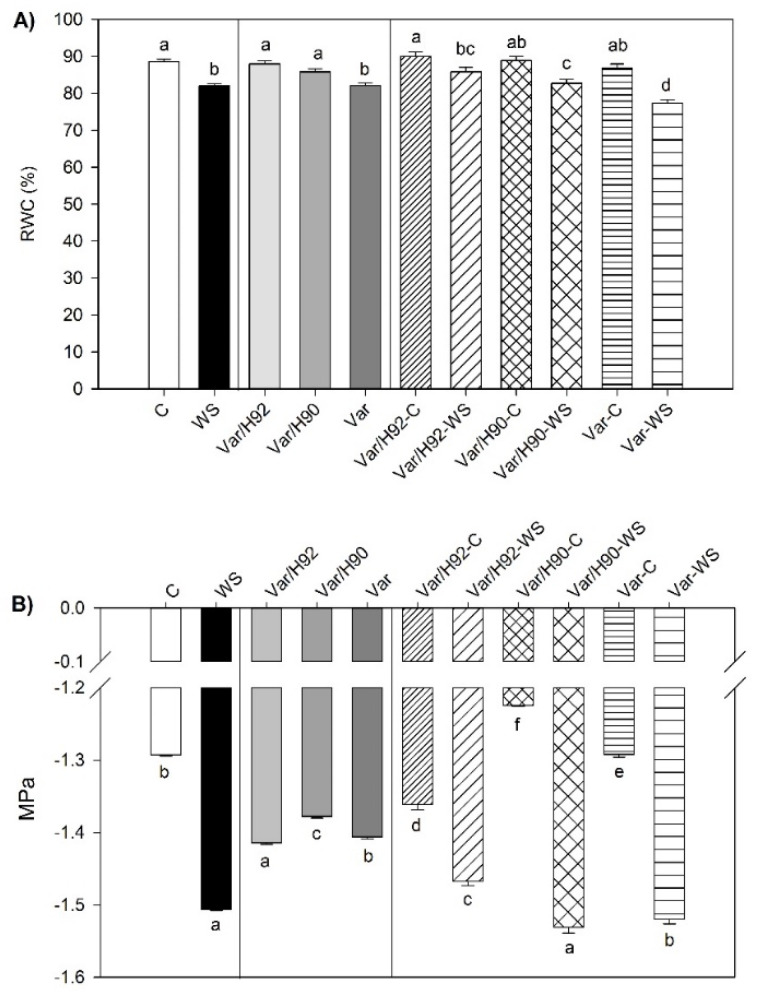
(**A**) Relative water content (RWC) and (**B**) osmotic potential (Ψs) in leaves for the ungrafted plants (Var, variety “Sueca”) and Var grafted onto H92 or H90 (Var/H92 and Var/H90, respectively) under the water stress (WS) or control conditions (C). Measurements were taken at 30 DAT (days after treatment). Data are the mean of four replicates and the error bars belong to the standard deviation for each plant type and treatment combination. Different letters indicate significant differences at *p* < 0.05 (least significance difference (LSD) test).

Leaf RWC values (Figure 1A) showed significant interaction between PT and T (*p* < 0.01). Plants under control conditions maintained RWC between 90 and 100% at the end of the experiment without significant differences among them. Water stress reduced significantly the RWC in all PT. Var plants were the most sensitive (77% RWC) followed by Var/H90 (83%) and Var/H92 (86%).

Leaf osmotic potential (Ψs) values at 30 DAT are showed in Figure 1B. Significant interaction was observed (PT × T) with *p* < 0.01. Under WS, all PT showed significant lower values compared to control conditions; Var/H90 displayed the largest decrease and Var/H92 the lowest drop in Ψs with significant differences among them.

### 3.2. Gas Exchange Measurements

All the measured photosynthetic parameters (except g_s_ at 30 DAT and A_N_/E at 20 DAT) exhibited a statistically significant interaction between treatment (T) and plant type (PT). For this interaction, A_N_ at 30 DAT, and g_s_ at 10 DAT presented *p* < 0.05, with *p* < 0.01 for the remaining photosynthetic parameters and DAT.

**Figure 2:**
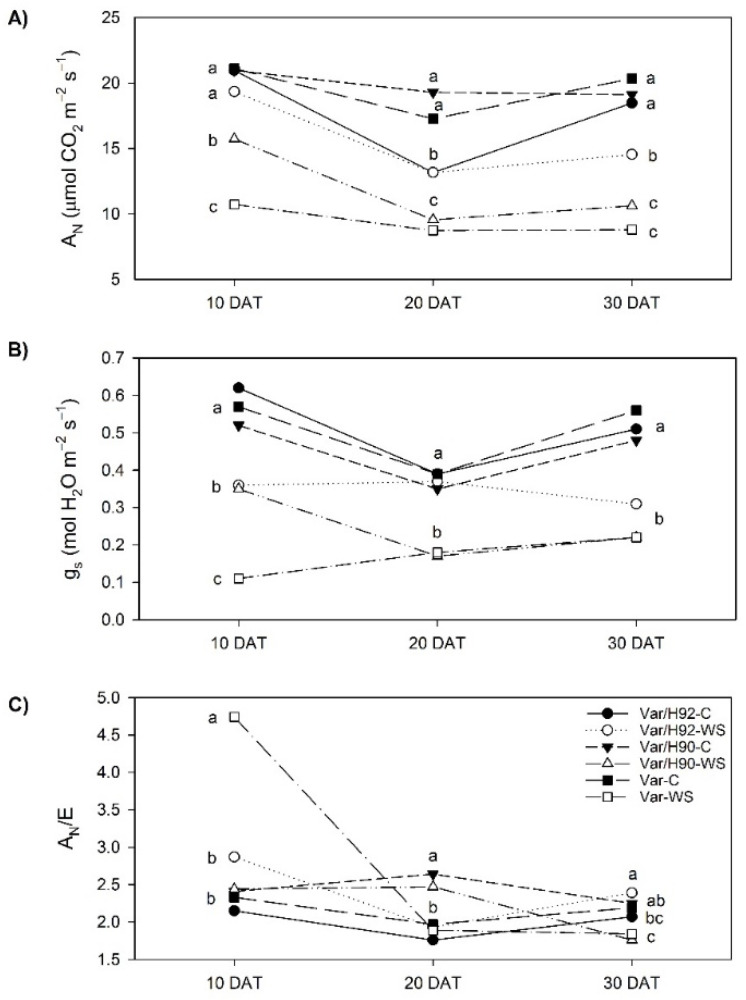
(**A**) CO_2_ assimilation rate (A_N_, µmol CO_2_ m^−2^ s^−1^), (**B**) stomatal conductance to water vapor (g_s_, mol H_2_O m^−2^ s^−1^), and (**C**) instantaneous water use efficiency (A_N_/E) in the ungrafted plants (Var, variety “Sueca”) and Var grafted onto H92 or H90 (Var/H92 and Var/H90, respectively) under the water stress (WS) or control conditions (C). Measurements were taken at 10, 20, and 30 DAT (days after treatment). Data are the mean value for *n* = 4 for each plant type and treatment combination. Different letters indicate significant differences at *p* < 0.05 (LSD test) for each measurement time separately.

The leaf CO_2_ assimilation rate (A_N_, Figure 2A) significantly lowered under water stress for the variety grafted onto H90 (Var/H90) and the ungrafted variety (Var) throughout the experiment. The variety grafted onto H92 (Var/H92) only displayed significant differences when comparing the control and water stress plants at the end of the experiment (30 DAT).

Leaf stomatal conductance (g_s_, Figure 2B) reduced in all the plant types during the experiment in the WS treatment compared to the control conditions. Only Var/H92 at 20 DAT did not show any significant differences between the control and stressed plants.

Instantaneous WUE (A_N_/E) did not reveal any significant differences at 20 DAT when the control and WS plants were compared for all the plant types (Figure 2C). At 30 DAT, all the plant types showed significant differences between treatments, Var/H92 increased A_N_/E, while Var/H90 and Var diminished A_N_/E under the WS conditions. Var also showed significant differences between treatments at the beginning of the experiment (10 DAT).

### 3.3. Ascorbic Acid Metabolism

For both the evaluated parameters, AsA (reduced ascorbate) and the AsA/AsA_t_ ratio, statistically significant interactions (PT × T) were observed throughout the experiment. AsA presented *p* < 0.01, while AsA/AsA_t_ showed *p* < 0.05 at 10 DAT and *p* ≤ 0.01 at 20 DAT and 30 DAT.

**Figure 3:**
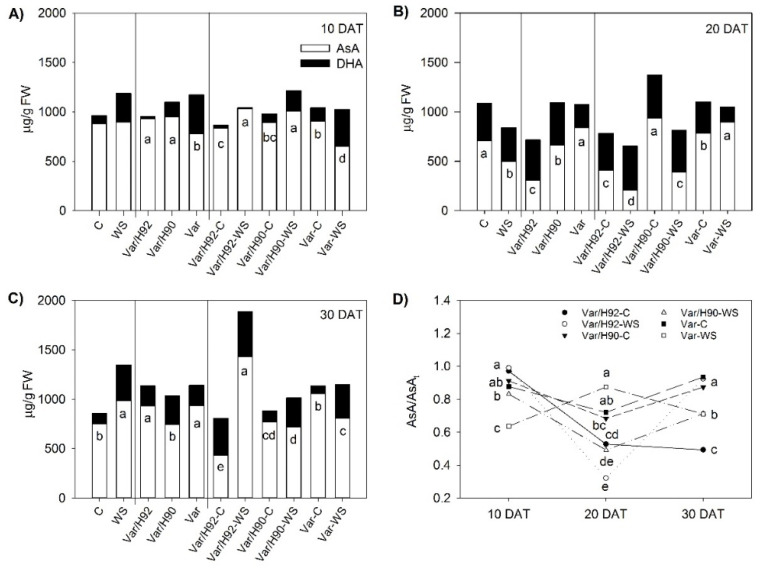
(**A**–**C**) Concentration of the different forms of ascorbate (AsA and DHA) in the leaves of the ungrafted plants (Var, variety “Sueca”) and Var grafted onto H92 or H90 (Var/H92 and Var/H90) under the water stress (WS) or control conditions (C) at 10, 20, and 30 DAT (days after treatment), respectively. Different letters indicate significant differences at *p* < 0.05 (LSD test) for the AsA parameter. No letters in 3A indicate no significant differences. (**D**) The AsA/AsAt ratio for the aforementioned combinations and time measurements. Different letters indicate significant differences at *p* < 0.05 (LSD test) for each measurement time separately. Data are the mean value for n = 4 for each plant type and treatment combination.

At 10 DAT, the AsA values under the WS conditions significantly differed from the C conditions for all the plant types (Figure 3A), with higher Var/H92 and Var/H90 values, and lower Var values. AsA/AsA_t_ only differed statistically in Var, with a lower ratio under the WS conditions (Figure 3D).

At 20 DAT, AsA showed significant differences for each plant type between treatments (Figure 3B). Var/H92 and Var/H90 dropped under water starvation, while Var rose. AsA/AsA_t_ (Figure 3D) also lowered in Var/H92 and Var/H90 under the WS conditions, while no significant differences appeared between treatments in Var.

Finally, at 30 DAT, AsA and AsA/AsA_t_ presented significant differences between treatments in Var/H92 and Var, whose values grew and fell, respectively, under WS (Figure 3C,D). Var/H90 only displayed differences for AsA/AsA_t_, which lowered under water starvation compared to the control plants (Figure 3D).

### 3.4. Catalase Activity

**Figure 4:**
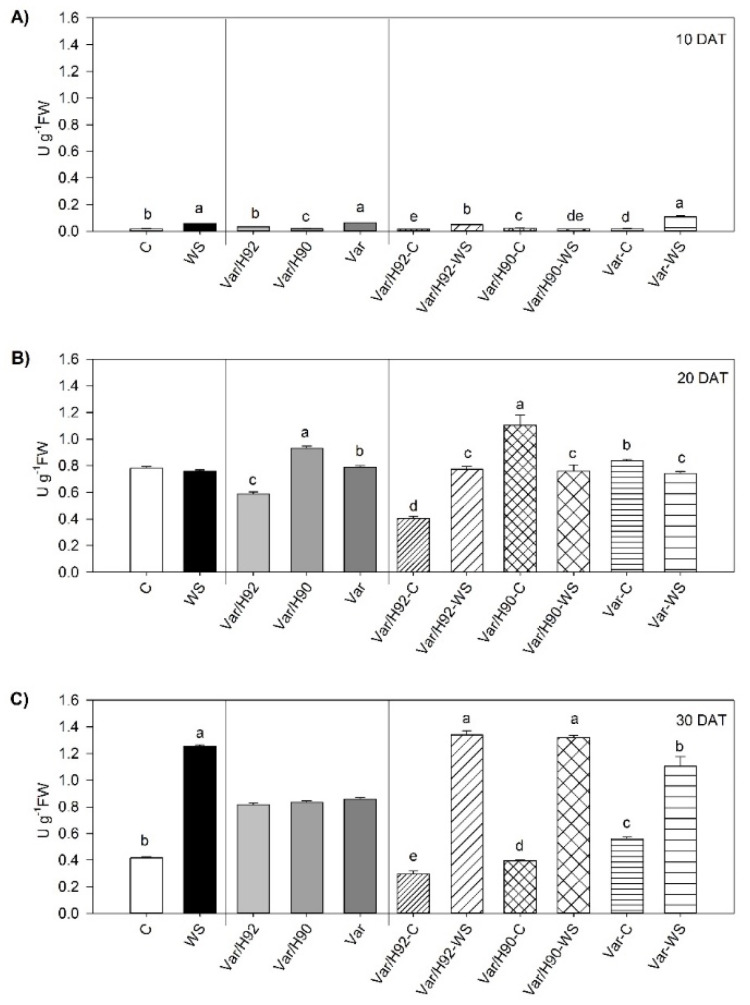
Catalase activity (as H_2_O_2_ reduction, U g^−1^ FW) in the leaves of the ungrafted plants (Var, variety “Sueca”) and Var grafted onto H92 or H90 (Var/H92 and Var/H90) under the water stress (WS) or control conditions (C) at 10 (**A**), 20 (**B**), and 30 (**C**) days after treatment (DAT). Different letters indicate significant differences at *p* < 0.05 (LSD test) for each measurement time separately. No letters in (**B**) an (**C**) indicate no significant differences. Data are the mean of four replicates and the error bars belong to the standard deviation for each plant type and treatment combination.

For this parameter, statistically significant interactions (PT × T) were found during the experiment (*p* < 0.01). All plant types showed significant differences between treatments while testing (Figure 4A–C).

Var/H92 significantly increased catalase activity during the experiment under WS (Figure 4A–C). In contrast, Var/H90 only boosted enzyme activity at the end of the experiment (30 DAT) under the water stress conditions, with reduced activity at 10 DAT and 20 DAT (Figure 4A–C). Lastly, Var obtained higher values when WS was applied at 10 DAT and 30 DAT, but lower ones at 20 DAT (Figure 4A–C).

### 3.5. Lipid Peroxidation

**Figure 5:**
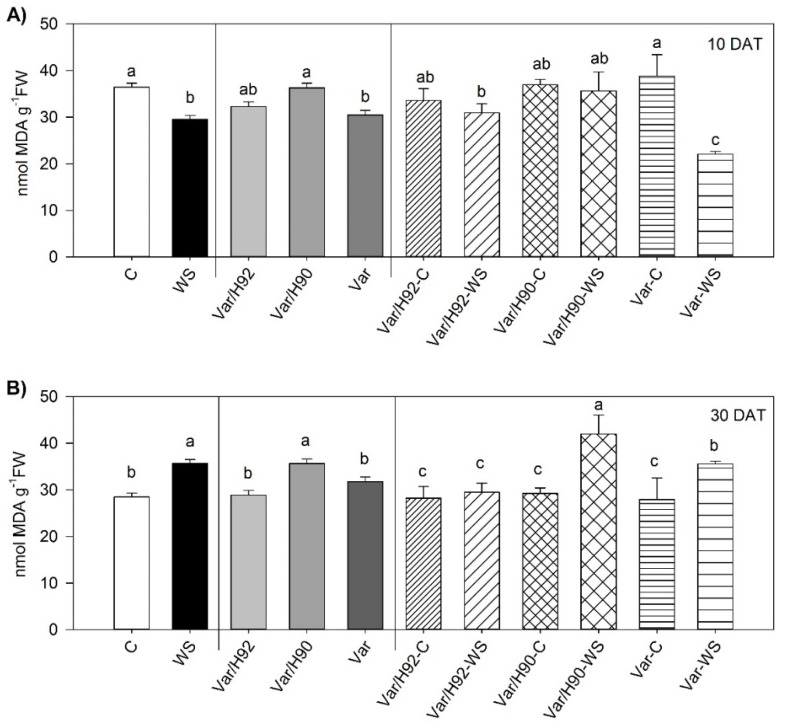
Lipid peroxidation (as malondialdehyde content, MDA) in the leaves of the ungrafted plants (Var, variety “Sueca”) and Var grafted onto H92 or H90 (Var/H92 and Var/H90) under the water stress (WS) or control conditions (C) at 10 (**A**) and 30 (**B**) days after treatment (DAT). Different letters indicate significant differences at *p* < 0.05 (LSD test) for each measurement time separately. Data are the mean of four replicates and the error bars belong to the standard deviation for each plant type and treatment combination.

Interactions for MDA content (*p* < 0.01) were found between T and PT throughout the experiment. Var/H92 appeared to be undisturbed when subjected to WS all along the experiment. At 10 DAT, only Var showed significant differences and the lowest MDA content for the stress conditions (Figure 5A). At 30 DAT, the MDA content of Var/H90 and Var rose significantly under WS (Figure 5B).

### 3.6. Proline Quantification

**Figure 6:**
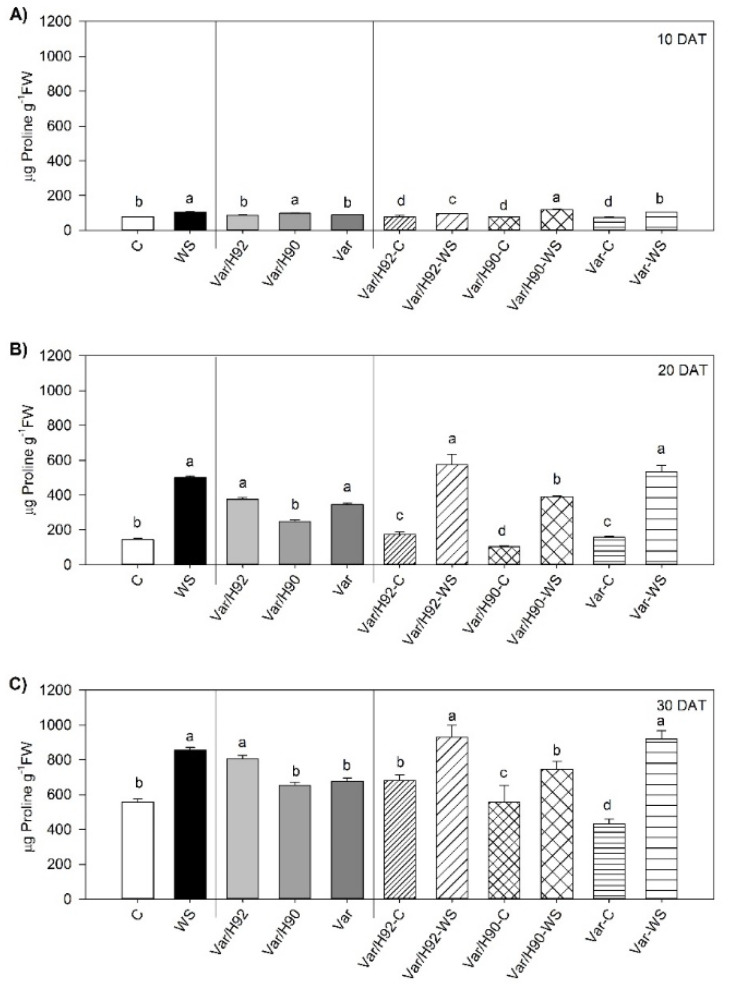
Proline content in the leaves of the ungrafted plants (Var, variety “Sueca”) and Var grafted onto H92 or H90 (Var/H92 and Var/H90) under the water stress (WS) or control conditions (C) at 10 (**A**), 20 (**B**), and 30 (**C**) days after treatment (DAT). Different letters indicate significant differences at *p* < 0.05 (LSD test) for each measurement time separately. Data are the mean of four replicates and the error bars belong to the standard deviation for each plant type and treatment combination.

Proline content showed interactions (*p* < 0.01) between PT and T while testing. The whole set of plants had a significantly higher proline content when water stress was applied throughout the experiment (Figure 6A–C). Notwithstanding, more marked increases took place for Var/H90 and Var at 10 DAT (Figure 6A), and also for Var/H92 and Var at 30 DAT (Figure 6B,C).

### 3.7. Biomass Determination

No significant interactions between T and PT were recorded for the measured biomass parameters (fresh and dry weight, leaf area, and number of leaves) (Table 1). 

The fresh weight of the plant aerial parts showed significant differences for both T and PT, with *p* < 0.01 and *p* < 0.05, respectively (Table 1). Aerial fresh weight reduced by 43% in the stressed plants vs. the control ones. The highest FW mean PT value was for Var/H92, followed by Var/H90 and Var (Table 1). 

For the dry weight of plant aerial parts, only T exhibited significant differences with *p* < 0.01. The plants under water stress had lower dry weight values than those under the control conditions (Table 1).

Leaf area exhibited significant differences for T and PT, with *p* < 0.01 and *p* < 0.05, respectively. Stressed plants reduced leaf area vs. the control plants. For PT, the biggest leaf area was for Var/H92, followed by Var and Var/H90 (Table 1). 

Significant differences were recorded only for number of leaves for T (*p* < 0.01). The plants under water stress had a smaller number of leaves than the plants under the control conditions (Table 1). 

## 4. Discussion

As expected, leaf RWC decreased under WS in all PT, but the highest reduction was in Var (22%) following Var/H90 (16%) and Var/H92 (12%), which showed the better RWC conservation. According to Hsiao [48], the decrease in RWC might indicate that Var/H90 and Var/H92 suffered a moderate WS while Var was affected by severe WS. The greater RWC in Var/H92 indicated higher capacity of water retention [49]. To maintain water uptake under the osmotic stress provoked by water scarcity, plants tend to decrease leaf Ψs. In our conditions, under WS, a fall in leaf Ψs was observed in all PT, and Ψs decrease was not enough to maintain RWC mainly in Var and Var/H90 plants. This drop in Ψs could be a consequence of a reduction in the leaf water content (dehydration) and/or due to active solute accumulation. Free proline is one of the most important osmolytes whose accumulation provokes a fall in Ψs [50]. Nevertheless, in our experimental conditions, the proline role contributing to lower Ψs was negligible, with mean values of 0.015 MPa and a 1.1% of contribution to Ψs, without ruling out that other components can be implicated in the reduction of osmotic potential [51].

Alterations in water relations under WS affected photosynthesis process in all PT. However, differential behavior was observed among them. After 10 DAT, A_N_ decreased in Var/H90 and Var, but not in Var/H92, which showed no significant differences with control plants. At the end of the experiment, A_N_ significantly decreased in all PT respect to their control, despite Var/H92 got the highest values under WS. These results agree with the ones obtained in grafted plants using tolerant rootstocks, which improved photosynthesis under WS (see reviews in [5,12]). In our experiment, the decrease in A_N_ showed linear correlation (*p* < 0.0001) with gs in all PT at 10, 20 and 30 DAT (r = 0.886, 0.742 and 0.937, respectively), which could mean that A_N_ reduction was mainly due to stomata limitations [25,30].

Grafting has been identified as an effective tool for increasing WUE in water stress situations [13,27]. A significant increase in WUE under water stress took place in Var/H92 at the end of experiment compared to the other PTs, which could result from the improvement in CO_2_ assimilation rate and the amelioration of stomata performance. In Var and Var/H90 in response to water stress, higher RWC reduction and Ψs can be related with stomatal closure and lower WUE, according to [52]. This result suggests fewer water requirements in Var/H92 combination, which is interesting because its water use should be lower. Similar results have been obtained by López-Marín et al., [17] in pepper plants grafted onto the “Terrano” rootstock, in cucumber grafted onto luffa [24] or in mini-watermelon grafted onto *Cucurbita maxima* × *C. moschata* [25]. It is noteworthy that our results showed how H92 rootstock could influence the scion-plant response to water stress in photosynthesis, stomatal conductance and WUE terms, which suggests that this plant combination performs better under water stress in gas exchange terms.

Despite the benefits of vegetable grafting on photosynthesis maintenance, and the antioxidant system role under water stress are both widely described in the scientific bibliography [5,12,53], few research has established a link between both plant mechanisms, especially in pepper grafted plants. Indeed, enhanced proline biosynthesis and proline degradation inactivation have been described to be involved in sustaining the electron flow between both photosystems by reducing photoinhibition and protecting the photosynthetic apparatus [54,55,56]. Accumulated proline also acts as a low-molecular-weight cellular antioxidant that protects plants from WS [57]. An improvement in proline accumulation under WS has been observed in grafted plants compared to ungrafted plants, and has been demonstrated in pepper [16] or tomato plants [49]. The increase in proline could be related to its protective role acting as a free radical scavenger, and also protecting thylakoids membrane and photosynthetic activity [56]; as it plays an important role in overcoming abiotic stress [50,56]. Indeed, a significant negative linear correlation between A_N_ and proline concentration (*p* < 0.01) was measured at 10 DAT, 20 DAT, and 30 DAT (r = −0.65, −0.72, and −0.52, respectively). This implies that while A_N_ decreased, proline increased with the same trend. This relation constituted a coupled response to water stress by minimizing its impact on plant performance, and it has been observed in pepper-grafted plants under salt stress [58] and sunflower [59]. The highest proline concentration herein observed was detected in Var/H92 and Var under WS from 20 DAT to 30 DAT. Nevertheless, a significantly lower photosynthesis rate was observed for the Var plants under WS, similarly to the Var/H90 plants. This finding indicates that other processes or antioxidant molecules apart from proline can be activated in Var/H92 plants to sustain photosynthesis under stress. A positive linear correlation (*p* < 0.01) was found at 10 and 30 DAT between A_N_ and AsA/AsA_t_ (r = 0.84 for 10 DAT and r = 0.55 for 30 DAT). Indeed, a marked significant increase in AsA and the AsA/AsA_t_ ratio was measured in Var/H92 at the end of experiment, when WS pressure on plants was higher. Higher AsA levels have been observed in tolerant tomato- and pepper-grafted plants under both salinity and water stress [8,60,61]. With stress, AsA is involved in protecting against ROS and photo-oxidative stress, that linked with sustained photosynthesis provides protection through the zeaxanthin interaction and subsequent thermal dissipation regulation [62,63]. No AsA increase was observed in the Var/H90 and Var plants under WS at 30 DAT. 

Plants under water stress overproduce ROS, one of them is H_2_O_2_, which is detoxified by catalase enzyme [64]. In our conditions, catalase activity significantly increased in all PT in response to WS, mainly at the end of the experiment. This increase was not consistent with the changes in lipid peroxidation, given that an important increase in lipid peroxidation was measured in Var/H90 and Var, but not in Var/H92. These results can indicate that other types of ROS could be implicated in the MDA production in Var and Var/H90, and the catalase activity was enough to eliminate H_2_O_2_ in Var/H92. 

Water-limiting conditions result in impaired growth, and reduce both the number of leaves and individual leaf size [65]. According to our results obtained under water stress, the fresh and dry weights of aerial plant parts, leaf area, and number of leaves decreased in all the plants respect to control conditions. In addition, DW values in aerial part did not show any significant differences between PT, probably because root biomass, which could be significant for total biomass, has not been quantified as the culture characteristics of potted plants with substrate made it difficult to obtain roots. 

Nonetheless, FW considering PT factor was higher in the Var/H92 plants, associated with higher water conservation or acquisition capacity. Leaf expansion normally depends on turgor pressure and assimilates supply. Thus, slow CO_2_ fixation could limit this process in Var/H90 and Var, and to a lesser extent in Var/H92 [65]. Nevertheless, our biomass values reflected the performance of whole plant net photosynthesis throughout the growth period, and not through the effect on the instantaneous net assimilation rate of CO_2_ by single-leaf measurements. 

## 5. Conclusions

In conclusion, the higher maintained rate of CO_2_ uptake observed using the tolerant rootstock H92 seems to involve minor oxidative stress, as observed by lesser membrane lipid peroxidation related to higher proline content and AsA concentration. Nonetheless, to validate these results, rootstock H92 should be tested under long-term experiments to evaluate its effects in terms of productivity and biomass under water stress.

## Figures and Tables

**Table 1:** Effect of factors treatment (T) and plant type (PT) on the fresh weight (FW) of aerial parts expressed as mean values and ANOVA.

		FW		DW		Leaf Area		Number of Leaves	
		(g Plant^−1^)		(g Plant^−1^)		(cm^2^)			
Treatment (T)									
C		335.3	a	50.27	a	6095	a	161.7	a
WS		191.4	b	28.88	b	3602	b	138.9	b
Plant Type (PT)									
Var/H92		277.6	a	41.15		5265	a	153.4	
Var/H90		267.6	ab	36.48		4469	b	143.3	
Var		244.9	b	41.09		4812	ab	154.3	
**ANOVA (*df*)**		**% Sum of Squares**							
T (1)		82.36	**	76.94	**	77.12	**	17.89	**
PT (2)		3.04	*	3.41		5.27	*	3.56	
T × PT (2)		2.09		1.64		4.45		10.07	
Residuals (30)		12.51		18.01		13.16		68.48	
Standard Deviation ^(+)^		30.6		5.64		594		24.3	

Different letters following the mean values indicate significant differences at *p* < 0.05 with the LSD test. An asterisk * indicates significant differences at *p* < 0.05 with the LSD test, while two asterisks ** indicate significant differences at *p* ≤ 0.01. ^(+)^ Calculated as the square root of the residual sum of squares. *df* degrees of freedom.

## Data Availability

The data presented in this study are available in the graphs and table provided in the manuscript.
